# Acceptability of and Implementation Supports for Video Directly Observed Treatment to Enhance Methadone Dosing Flexibility in a Multisite Opioid Treatment Program: Qualitative Rapid Needs Assessment Study

**DOI:** 10.2196/84162

**Published:** 2026-01-12

**Authors:** Judith I Tsui, Elizabeth J Austin, Julia A Dunn, Alexander J Gojic, Elenore P Bhatraju, James Darnton, Paul Grekin, Sean Soth, Steve Woolworth, Emily C Williams, Kevin A Hallgren

**Affiliations:** 1 Division of General Internal Medicine Department of Medicine University of Washington Seattle, WA United States; 2 Department of Health Systems and Population Health School of Public Health University of Washington Seattle, WA United States; 3 Evergreen Treatment Services Seattle, WA United States; 4 Department of Psychiatry and Behavioral Sciences School of Medicine University of Washington Seattle, WA United States; 5 Health Services Research & Development Center of Innovation for Veteran-Centered and Value-Driven Care Veterans Affairs Puget Sound Health Care System Seattle, WA United States

**Keywords:** opioid use disorder, methadone, addiction treatment, mobile health, mHealth

## Abstract

**Background:**

Methadone is a first-line treatment for opioid use disorder, which is delivered in federally regulated opioid treatment programs (OTPs). Federal policies require directly observed dosing of methadone followed by graduated provision of nonobserved doses to take at home (ie, “take-home” dosing) after demonstrated stability is achieved. Policy changes since the COVID-19 pandemic have greatly expanded take-home dosing. Video directly observed treatment (video DOT) is an approach in which patients submit videos of themselves taking medications, which are asynchronously reviewed to verify adherence.

**Objective:**

In preparation for an implementation trial evaluating the adoption of video DOT in OTP settings, we conducted a rapid needs assessment with multidisciplinary stakeholders to assess acceptability, perceived benefits, and needed support for video DOT to monitor take-home methadone dosing.

**Methods:**

In our rapid needs assessment, we explored perspectives of multidisciplinary stakeholders (N=20) at 3 clinical sites within a single OTP in western Washington state. Trained qualitative researchers took ethnographic field notes during meetings with organizational leadership and in-person site visits with clinical and administrative staff. Field notes were analyzed via a team-based rapid assessment process using coding templates informed by the Consolidated Framework for Implementation Research. Summaries of qualitative data were iteratively reviewed by the study team and further confirmed with site stakeholders.

**Results:**

Stakeholders included leadership (n=6, 30%), medical providers (n=4, 20%), substance use disorder counselors (n=7, 35%), and clinic managers and support staff (n=3, 15%). Stakeholders perceived that video DOT could lessen the barriers patients face, including travel burden (eg, time and cost) and stigma. They also identified that video DOT could have important impacts on early care retention, given expansions of take-home dosing. However, stakeholders anticipated an added burden for clinical staff and emphasized the need for implementation supports that would limit burden, such as additional staff support for video submission review and clear communication pathways when video submissions require additional clinical input.

**Conclusions:**

A rapid needs assessment of OTP sites for a future implementation study suggested that stakeholders saw potential benefits for patients receiving video DOT, but there were concerns that this would add to their work burden. Learnings informed the subsequent tailoring of clinical use cases and implementation supports.

## Introduction

Opioid use disorder (OUD) remains a major cause of morbidity and mortality in the United States. As of 2024, an estimated 4.5 million American adults had OUD [1]. Since 2008, opioid overdose has exceeded motor vehicle accidents as a cause of death of adults [2], and mortality rates have markedly increased with the emergence of fentanyl as the prevailing opioid used, with the highest rates of increase seen among Black American, Native American, and Hispanic or Latinx American individuals [3]. Although there has been a recent downward trend in fatal overdose events nationally, not all parts of the country have witnessed declines, and the absolute number of overdose deaths—87,000 from 2023 to 2024—remains unacceptably high [4]. Opioid use is linked to recent outbreaks of HIV and increased incidence of hepatitis C virus among young adults in both rural and urban areas of the United States [5-8]. In addition, untreated OUD is a major burden on society, leading to excess health care use, loss of work productivity, crime, and incarceration [9].

Methadone was the first medication approved for OUD, has been a cornerstone of treatment for decades, and is associated with reductions in opioid use [10] and related mortality [11]. The arrival of fentanyl has posed challenges with initiating buprenorphine due to the risk of precipitated withdrawal, which has led to renewed interest in methadone as a treatment modality for OUD among patients and health care providers. In the United States, daily or frequent supervised treatment with visual confirmation of ingestion (directly observed therapy; DOT) is the standard of care for methadone treatment in federally licensed opioid treatment programs (OTPs). DOT mitigates the risk of medication poisonings and diversion; however, these same policies present major challenges for many clients due to potentially long travel times; interference with other obligations (eg, work, school, and family responsibilities); perceived stigma; and lost autonomy stemming from the requirement of frequent in-person visits, which are not required for almost all other medications.

In response to the COVID-19 pandemic, the Substance Abuse and Mental Health Services Administration released adjusted rules governing OTPs, allowing states to request blanket exceptions for all “stable” clients to receive 28-day take-home dosing and for “less stable” clients to receive 14-day take-home dosing if OTP medical providers believe that this level of take-home dosing could be safely handled [12]. The evidence to date suggests that relaxing regulations during the COVID-19 pandemic resulted in OTPs granting more take-home methadone doses without a substantive increase in methadone-involved overdose [13], and this change was recently codified as part of 42 Code of Federal Regulations Part 8 [14]. However, clients and health care providers also articulate drawbacks from the loss of structure and accountability from DOT [15]. In addition, with the loss of strictly codified algorithms for take-home doses, a greater weight of decision-making is transferred to the subjective assessments of OTP medical providers. Given shifting drug supplies and increases in fentanyl and methamphetamine use (which are associated with fatal overdose trends), health care providers are increasingly burdened with finding the right balance between flexibility and convenience and safety in a setting where there are high stakes and low tolerance for errors in medication taking.

Video directly observed therapy (video DOT) is an approach in which patients submit videos of themselves taking medications, which are asynchronously reviewed to verify adherence. Such an approach may be a middle ground in obviating the need for in-person visits while retaining the benefits of confirmed ingestion. We conducted a clinical pilot during the COVID-19 pandemic that demonstrated the feasibility of implementing video DOT via smartphone app for methadone dosing [16-18]. The pilot study also found that the video DOT app was associated with higher rates of confirmed doses; compared to matched control clients in the same setting, clients who used video DOT demonstrated higher rates of observed dosing (eg, mean 53.2 days vs 16.6 days with an observed dose over the first 60 days [17]) and higher rates of “graduating” to an increased number of take-home doses within the first 60 days (61% vs 0%). While multiple previous usability studies, including our own, have found app-based technologies acceptable for take-home methadone dosing [19-21], few studies have explored which implementation supports are needed to scale these technologies across the clinic. Implementation supports that enable and promote adoption of new technologies in clinical practice often need to be tailored to the local context, highlighting the benefit of formative evaluation in the early stages of implementation research [22,23].

Evidence that video DOT can be implemented in multiple OTPs can inform policies and reimbursement mechanisms that support video DOT as a means to achieve more flexible, client-centered models of methadone treatment. In preparation for an implementation trial evaluating the adoption of video DOT in OTP settings, we explored stakeholder perspectives on acceptability, perceived benefits, and needed supports for video DOT implementation to monitor take-home methadone dosing as part of the needs assessment conducted during the preimplementation phase.

## Methods

### Study Design Overview

As a component of formative evaluation activities for our hybrid type 2 effectiveness-implementation study that tested implementation and clinical outcomes for video DOT for methadone across a multisite OTP, we conducted a rapid needs assessment with multidisciplinary stakeholders at 3 implementation sites to prepare for implementation.

### Intervention Description

The video DOT technology involves (1) a patient-facing app, used on smartphones to submit routine videos of their methadone dosing, and (2) a web-based portal for clinical staff that allows staff to manage patient enrollment, monitor adherence, and message patients directly about video submissions and other issues. To initiate a patient’s use of video DOT, clinical staff need to create an account for them and provide information about their methadone dosing (eg, dose amount and timing), assist the patient in downloading the app to their phone, and provide any additional training on navigating the app. Once enrolled, patients receive daily reminders to take their dose and instructions within the app on how to submit their videos (including showing their dosing bottle and verifying dose ingestion). Videos can be recorded offline and submitted for review once patients have access to Wi-Fi or cellular data. Patients are also able to use messaging functions and monitor their adherence via a tracking calendar. Video submissions are reviewed by members of the team to assess whether they meet criteria for acceptance and provide patient education as needed. Clinical teams are alerted to any clinical issues or missed video doses via email and web portal notifications. Clinical teams can also log into the web portal to review patient, caseload, and clinic-level metrics on enrollment and adherence in real time. When a patient has completed their use of video dosing, clinical staff can deactivate their account in the web portal.

### Study Population and Setting

The study setting is a large OTP serving more than 2500 clients at 3 sites in cities in western Washington state (ie, Seattle, Renton, and Olympia). The OTP serves a broad swath of patients from those cities as well as surrounding suburban and rural areas (>300 zip codes). The organization played a leading role in developing procedures to respond to the COVID-19 pandemic, culminating in a revision to federal policy to relax requirements for supervised DOT ingestion [12]. The organization serves a high percentage of clients who are unhoused or unstably housed and vulnerable to disruptions in care as well as individuals from diverse racial or ethnic backgrounds [17,24].

For the rapid needs assessment, we engaged clinical stakeholders across the 3 clinical implementation sites. Stakeholders included organizational leaders, clinic managers, medical providers (all physician assistants and advanced registered nurse practitioners), behavioral health providers (ie, counselors), and other staff.

### Data Collection

In the preimplementation phase of the study, we conducted 3 site visits (1 per site) and held a series of meetings with organizational leadership to discuss program needs and goals for video DOT. During all site visits and meetings, we engaged in participant observation and data collection in the form of ethnographic field notes. Site visit discussions followed a semistructured discussion guide that asked staff to reflect on how video DOT might support methadone care delivery and what implementation approaches would be most supportive. Site visits also involved informal discussions with staff while on site, in accordance with ethnographic data collection methods [25-28]. Trained qualitative researchers (EJA, ECW, and JAD) took ethnographic field notes consisting of near-verbatim typed notes documenting the contents (eg, conversations and actions) of all implementation meetings and site visit discussions. This is an ethnographic method of data collection that is recommended in formative evaluation [23,26,29], which our team has used in several previous studies to understand barriers and facilitators in real time and address barriers with adaptive implementation strategies that capitalize on facilitators [30-32]. The rapid needs assessment meetings with health care providers and clinical staff members were facilitated by research team members (JIT, KAH, EJA, ECW, and JAD) to develop a shared and more precise understanding of (1) program-level challenges with integrating video DOT into clinical workflows (including time requirements for staff to review videos and respond to messages from the application about missed videos), (2) attitudes and concerns related to video DOT, (3) how to identify clients for whom video DOT is appropriate, (4) expectations for how video DOT can lead to fewer in-person visits and more take-home doses with the ultimate goal of accelerating the timeline to unobserved take-home doses, (5) whether to use add-on features that might enhance monitoring or case management (including, but not limited to, appointment reminders; 2-way chat; and links to community resources, such as food banks, syringe service programs, housing resources, and COVID-19 testing), and (6) when to discontinue video DOT (both criteria for discontinuation and completion of video DOT) and any changes to clinical care that may be needed if video DOT is discontinued (eg, changes in take-home frequencies). The results of the rapid needs assessment informed training and practice support materials, workflow diagrams, case examples, and decision-support guidance in the implementation period.

### Analysis

Ethnographic field notes were analyzed by formative evaluation team members with qualitative analysis expertise (EJA and JAD) using a rapid assessment process (RAP), an intensive, team-based qualitative inquiry process that uses triangulation, iterative data analysis, and additional data collection to develop an understanding of a situation from the insider’s perspective [29,33-35]. Consistent with our previous research using the RAP [30-32], data from all notes from participant observations were reviewed iteratively and coded in structured templates guided by the domains of the Consolidated Framework for Implementation Research: characteristics of the intervention and individuals using it, the outer and inner settings in which implementation occurs, and the implementation process [36]. In each of these 5 broad domains of the Consolidated Framework for Implementation Research, there are multiple subdomains; together domains can be used to guide implementation efforts and identify strategies for implementation in post hoc analysis. Analyses involved a combination of deductive and inductive approaches that allowed for emergent themes that characterized potential influencing factors and needed supports for the implementation. Analyses were conducted iteratively and shared with the entire investigative team for further interpretation.

### Ethical Considerations

The University of Washington Institutional Review Board provided oversight and approval for this research (STUDY00011142). For collection of the ethnographic field notes described above, the institutional review board determined the informed consent process could be waived and that compensation for participation was not required. To maintain privacy and confidentiality of staff members, neither demographic nor identifiable information were collected as a part of the ethnographic field notes.

## Results

### Overview

Across the 3 sites, 20 individuals participated in preimplementation discussions, of which 8 (40%) were frontline clinical care providers, 9 (45%) had a supervisory administrative role, and 3 (15%) had a leadership role in the organization. Among clinical care providers, 8 (40%) were counselors, 8 (40%) were medical providers (who were advanced practice providers, either nurse practitioners or physician assistants), and 4 (20%) had nursing or other roles. Among the 3 sites, the average patient census was estimated to be approximately 900 (range 500-1250).

A total of 4 themes that emerged from analyses of the data are detailed subsequently and summarized in Table 1. These themes characterized perspectives on using video DOT in routine practice and contributed to the design and tailoring of implementation supports. Through discussions, several case use scenarios for video DOT emerged; these are summarized in Table 2 along with potential outcomes to measure the success of use.

**Table 1:** Summary of themes and implementation team response.

Needs assessment theme	Implementation team response
Perceived benefit for patients facing access burdens	Expanding video DOT^a^ eligibility criteria to prioritize patients with high access burdens Revising use cases to accommodate additional clinical scenarios that patients with high access burdens commonly face
Potential tool to help clinical teams respond to changing guidelines and drug supply	Developing potential secondary use cases that may be piloted (eg, use of video DOT to help reach therapeutic dose, buprenorphine tapering, and callbacks to verify medication supply)
Need to center implementation on ease of use and team-based communication	Identifying and preparing multidisciplinary champions at each site Co-designing implementation support materials (training and workflows) Incorporation of local team-based communication modalities
Challenge of balancing harm reduction and safety in video DOT use	Co-design of protocols for video DOT monitoring and notification of clinic staff for unexpected issues (escalation) Use of team-based communication strategies for escalation scenarios Ongoing audit and feedback

^a^Video DOT: video directly observed treatment.

**Table 2:** Summary of potential video directly observed treatment (video DOT) use cases and goals.

Stage of care	Potential video DOT use case	Outcomes of interest
Treatment initiation	Monitor initial methadone dose titration	Shortened time to reach therapeutic dose Improved adherence or reduction in missed doses
Treatment monitoring	Provide additional support for patients increasing take-home doses Enable virtual callbacks for patients on take-home doses Provide encouragement or positive reinforcement to patients for consistent engagement or adherence (eg, through asynchronous video or SMS text messages from the care team) Provide additional structure for patients needing short-term exceptions (eg, an unexpected need for take-home doses) Provide monitoring for patients with split dosing	Increased take-home doses per patient Improved MOUD^a^ adherence and days of coverage (ie, fewer missed doses) Improved engagement and retention in care Reduced diversion or misuse of methadone Reduced illicit opioid and polysubstance use Reduced time and cost associated with transportation and access burden for patients Improve recognition and responsiveness to patient health needs (eg, communication about side effects and dosing issues) Increased patient and health care provider satisfaction with care delivery
Treatment completion and transition to the next phase of care	Monitor transition to buprenorphine or other MOUD	Shortened time to reach therapeutic dose

^a^MOUD: medications for opioid use disorder.

### Theme 1: Video DOT May Be Most Beneficial for Patients Facing Structural Barriers to Accessing the Clinic

As clinical teams considered adding video DOT to their practice, they identified many potential clinical and patient-centered benefits of video DOT across the OUD care continuum (Table 2). They acknowledged that some patients experienced heightened structural barriers to accessing the clinic and may therefore benefit more greatly from video DOT. For example, participants shared that many patients travelled long distances to get to the clinic. One participant shared that their clinic covered patients across the 5 neighboring counties, a large geographic region. Another participant described that their clinic was only accessible by 1 bus route, making public transportation to the clinic arduous. One participant shared the following:

Transportation is a lot of our big issues, because we cover a lot and we’re it, it’s a real struggle for some of them to get here.

Participant A15

Another echoed this, saying the following:

I see this as something really positive, it’s so much [less] stress for them to not have to travel or take time off work.

Participant A20

Participants also articulated that video DOT “might be helpful for folks with mobility issues” (participant A18). Participants described that patients with mobility issues or physical disabilities often struggled to get to the clinic. One participant said the following:

Just the disability…this would really serve that population.

Participant A16

Overall, participants believed that video DOT might be suitable for some but not all patients, which would still make it a helpful tool. One participant expressed the following:

If you help one person, it’s worth it.

Participant A16

### Theme 2: Video DOT May Help Clinical Teams Be More Responsive to How Methadone Treatment Delivery Is Rapidly Evolving

Participants described how shifting drug supply and the revision of federal policies governing methadone treatment during the COVID-19 pandemic have changed the landscape of day-to-day care for OUD treatment with methadone. For example, the practice of take-home dosing has expanded rapidly, such that daily in-person clinic dosing is *the exception not the rule now* (participant A8). While participants viewed the shift to increased take-home doses positively, they also felt challenged by maintaining patient engagement in care in the context of fewer in-person clinic interactions. Participants considered video DOT a useful bridge for facilitating patient engagement when fewer in-person dosing visits were happening:

Can we show that we have better engagement and retention?...if we get better engagement then it pays for itself.

Participant A9

Similarly, another participant shared that video dosing may help clinical staff trying to manage increased caseloads, saying the following:

As caseloads grow, [clinical staff] are getting very frustrated...this might be an option that encourages better attendance.

Participant A16

Participants also described that increasing fentanyl use among patients has been a challenge for initiating methadone. Fentanyl is a highly potent synthetic opioid that quickly leads to high physical dependence. It may also be retained in adipose tissues, leading to physiological persistence. As a result, severe and prolonged withdrawal may occur, necessitating higher doses of methadone to control cravings and withdrawal symptoms. One participant shared the following:

Just with fentanyl use, it’s made everybody’s dose much higher.

Participant A4

Rapidly achieving higher doses of methadone to adequately treat fentanyl withdrawal is accompanied by risks for oversedation (and related complications, such as accidents and falls). Participants viewed video DOT as a potential tool to help them find that balance between rapid dose escalations and the need to monitor in-person for safety. One participant shared the following:

Especially if you didn’t have to connect with the provider, that would be an amazing tool because we’re constantly talking about how we can get people to higher doses quicker.

Participant A9

### Theme 3: Implementation of Video DOT Needs to Center on Ease of Use and Team-Based Communication Strategies

When reflecting on using video DOT in routine practice, participants worried that it might add burden to their day-to-day workload. Participants expressed that video DOT needed to be easy to use to ensure uptake among busy team members:

We do like simple.... The counselors get loaded down with a lot.

Participant A16

As part of this, participants emphasized the importance of having clear and inclusive communication pathways about video DOT use by their patients. Specifically, participants reflected that communication about any patient issues with video DOT needed to involve the entire clinical team (ie, medical, counseling, and nursing) to support team-based decision-making. Yet, communication also needed to be clear on which team member is accountable for subsequent actions:

My concern over shared accountability is that there is no accountability. So having a designated person and maybe depending on the case allowing someone to delegate.... I want to say “[team member] that was your job.”

Participant A9

Although there was a desire for clear workflows and accountability related to video DOT use, participants were reluctant to develop rigid policies regarding video DOT eligibility and adherence thresholds, given that each patient and clinic may have unique scenarios to consider. One participant further expressed that it “might be nice to see how [video dosing] functions differently in different settings because we have vastly different populations” in each clinical site (participant A9).

Finally, there was a shared consensus that the primary goal of leveraging video DOT should be to increase take-home dosing for patients who might not otherwise qualify. As one participant described, video dosing should prioritize patients who are “really on the fence or folks that are on the bubble [that] we could get to take-homes faster” (participant A9); another participant shared that video dosing would be ideal for “that patient [who] is stuck at [daily dosing] for a while and I know they really should be on monthlies” (participant A4).

### Theme 4: Balancing Harm Reduction and Safety May Be a Challenge in Video DOT Use

Participants noted that their perspectives on harm reduction have changed in response to changing policies regarding take-home dosing. One participant shared the following:

We are less restrictive than we use to be.... If you walk in the door, we will do what we need to do to get treatment.

Participant A12

Another participant described the following:

The take-home policy is so vastly different than it was the first time around and the real focus is safety. It’s really a safety lens for daily dosing.

Participant A10

Participants identified that using video DOT in routine practice may introduce new, unforeseen scenarios related to patient safety and diversion. One participant shared an example related to diversion, reflecting on the need to identify patients who had the best chance of being successful with video DOT:

We talked originally about diversion...there’s not a ton we can do. There’s ways to have the tamperproof bottles...so we can have confidence [that the patient has taken the correct dose]. It’s really about selecting a population that’s going to benefit from this and that it will be meaningful for.

Participant A9

Other participants discussed the need to have ongoing discussions and implementation adaptations to address evolving needs to support patient safety, which is a priority within the culture of the organization. One participant summarized, their organization “has always erred on the side of caution” (participant A15).

## Discussion

This rapid needs assessment of 3 clinic sites within a large OTP in western Washington was conducted during the preimplementation phase of a planned hybrid effectiveness-implementation study and offered valuable insights from stakeholders on anticipated acceptability, benefits, and needed supports for implementing video DOT to monitor take-home methadone dosing. A key theme was the belief that video DOT can address several access barriers patients face, including travel burden (both in terms of time and cost) and mobility or physical disability issues. Participants also perceived value in how video DOT may help clinical teams adapt to new expectations for methadone care delivery, namely the expansion of take-home dosing and more rapid up-titration of dosages, as well as its potential in helping clinical teams strike the right balance between a risk reduction approach and ensuring patient safety. Stakeholders emphasized the need for implementation support that would limit burden for clinical staff, such as additional staff support for video submission review and clear communication pathways for when video submissions require additional clinical input. Findings from this work subsequently guided the tailoring of implementation supports—including training and practice support materials, workflow diagrams, case examples, and decision support guidance for video DOT for methadone—that were co-designed with OTP stakeholders in preparation for the study’s launch of video dosing.

This study builds on previous research demonstrating the feasibility of video DOT for methadone, including an observational study that showed video DOT increases rates of observed dosing and “graduation” to increased take-home doses within the first 60 days of use [17], as well as usability studies showing that patients and counselors are able to use the video DOT app correctly [16]. The research is timely, as many of the federal policy changes for OTPs implemented during the COVID-19 pandemic have been made permanent or extended to increase access to treatment for OUD [14]. Patients on methadone may benefit from expansion of this intervention, considering the myriad challenges these patients face with expectations for in-person dosing with regard to costs, travel time, exposures to drug activities, and stigma. Evidence that video DOT can be implemented in OTPs may help actualize the end goal of such policies that strive to ensure more flexible, client-centered methadone delivery. While federal policy made it easier for patients to receive take-home doses, heterogeneity in practice exists in part due to variability in approaches among clinics and health care providers [37]. Thus, there is still an urgent need to create systems of care delivery that will facilitate more take-home doses and reduce the need for travel to clinic visits [38]. It is notable that our discussions with stakeholders revealed other potential use case scenarios beyond advancing take-home doses, such as monitoring patients on split dosing or rapid dose escalation. We anticipate that future research will provide additional valuable information on how this technology can be feasibly adapted to best serve patients and health care providers.

There are limitations to the generalizability of findings from this RAP. While we attempted to draw in as many provider stakeholders as possible at the sites, the findings do not represent all perspectives or opinions. Similarly, our focus on a single OTP may limit generalizability. The rapid needs assessment process relied on qualitative ethnographic methods intended to capture an in-depth understanding of a local context. As the goal of the research was to quickly obtain an understanding from an insider’s perspective to guide implementation efforts, we felt this was the most expeditious approach. However, further methods—particularly those that capture quantitative and additional qualitative methods with expanded samples—would support a better understanding of the implementation context and outcomes for video dosing use in OTP settings. We will examine these issues as part of the ongoing implementation trial at the single OTP in which we are working.

In summary, a rapid needs assessment of OTP sites for a future implementation study suggested that stakeholders saw potential benefits for patients with video DOT for methadone, but there were concerns that this would add to staff work burden. In the context of in-person dosing becoming the exception instead of the norm, stakeholders perceived video DOT as being a potentially useful tool for a subset of patients and mainly to facilitate advancement of take-home doses, and additional unique case scenarios for video DOT use were identified. The findings inform our next steps in implementing video DOT for methadone in the OTP clinic sites and measuring the success of implementation and its impact on clinical outcomes.

## Abbreviations

DOT: directly observed treatment
OTP: opioid treatment program
OUD: opioid use disorder
RAP: rapid assessment process
Video DOT: video directly observed treatment

## Data Availability

The datasets generated or analyzed during this study are available from the corresponding author on reasonable request.
